# A Multiplex Test Assessing *MiR663a*_me_ and *VIM*_me_ in Urine Accurately Discriminates Bladder Cancer from Inflammatory Conditions

**DOI:** 10.3390/jcm9020605

**Published:** 2020-02-24

**Authors:** Sara Monteiro-Reis, Ana Blanca, Joana Tedim-Moreira, Isa Carneiro, Diana Montezuma, Paula Monteiro, Jorge Oliveira, Luís Antunes, Rui Henrique, António Lopez-Beltran, Carmen Jerónimo

**Affiliations:** 1 Cancer Biology and Epigenetics Group—Research Center (CI-IPOP), Portuguese Oncology Institute of Porto (IPO Porto), and Porto Comprehensive Cancer Center (P.CCC), Maimonides Biomedical Research Institute of Cordoba, 14004 Cordoba, Spain; sara.raquel.reis@ipoporto.min-saude.pt (S.M.-R.); joana.matos@ua.pt (J.T.-M.); isa.carneiro@ipoporto.min-saude.pt (I.C.); dianafelizardo@gmail.com (D.M.); paula.monteiro@ipoporto.min-saude.pt (P.M.); henrique@ipoporto.min-saude.pt (R.H.); 2 Department of Urology, University Hospital of Reina Sofia, Maimonides Biomedical Research Institute of Cordoba, 14004 Cordoba, Spain; anblape78@hotmail.com; 3 Department of Pathology, Portuguese Oncology Institute of Porto (IPO Porto), 4200-072 Porto, Portugal; 4 Department of Urology, Portuguese Oncology Institute of Porto (IPO-Porto), 4200-072 Porto, Portugal; jorge.oliveira@ipoporto.min-saude.pt; 5 Department of Epidemiology, Portuguese Oncology Institute of Porto (IPO Porto) & Cancer Epidemiology Group—Research Center (CI-IPOP), 4200-072 Porto, Portugal; luis.antunes@ipoporto.min-saude.pt; 6 Department of Pathology and Molecular Immunology, Institute of Biomedical Sciences Abel Salazar (ICBAS)—University of Porto, 4050-313 Porto, Portugal; 7 Department of Surgery and Pathology, Faculty of Medicine, University of Cordoba, 14071 Cordoba, Spain; em1lobea@uco.es; 8 Champalimaud Clinical Center, 1400-038 Lisbon, Portugal

**Keywords:** bladder cancer, methylation, biomarkers

## Abstract

Bladder cancer (BlCa) is a common malignancy with significant morbidity and mortality. Current diagnostic methods are invasive and costly, showing the need for newer biomarkers. Although several epigenetic-based biomarkers have been proposed, their ability to discriminate BlCa from common benign conditions of the urinary tract, especially inflammatory diseases, has not been adequately explored. Herein, we sought to determine whether VIM_me_ and miR663a_me_ might accurately discriminate those two conditions, using a multiplex test. Performance of VIM_me_ and miR663a_me_ in tissue samples and urines in testing set confirmed previous results (96.3% sensitivity, 88.2% specificity, area under de curve (AUC) 0.98 and 92.6% sensitivity, 75% specificity, AUC 0.83, respectively). In the validation sets, VIM_me_-miR663a_me_ multiplex test in urine discriminated BlCa patients from healthy donors or patients with inflammatory conditions, with 87% sensitivity, 86% specificity and 80% sensitivity, 75% specificity, respectively. Furthermore, positive likelihood ratio (LR) of 2.41 and negative LR of 0.21 were also disclosed. Compared to urinary cytology, VIM_me_-miR663a_me_ multiplex panel correctly detected 87% of the analysed cases, whereas cytology only forecasted 41%. Furthermore, high miR663a_me_ independently predicted worse clinical outcome, especially in patients with invasive BlCa. We concluded that the implementation of this panel might better stratify patients for confirmatory, invasive examinations, ultimately improving the cost-effectiveness of BlCa diagnosis and management. Moreover, miR663a_me_ analysis might provide relevant information for patient monitoring, identifying patients at higher risk for cancer progression.

## 1. Introduction

Bladder cancer (BlCa) is one of the most incident cancers, ranking ninth in prevalence worldwide [1,2]. In men, which are more prone to develop BlCa, it represents the second most frequent urological malignancy after prostate cancer [1,2]. Moreover, it is expected that, by 2040, the number of estimated new cases and cancer-related deaths will almost double the 549,393 newly diagnosed cases and 199,922 deaths recorded in 2018 [1,2]. Most BlCa cases correspond to urothelial carcinoma, generally presenting as non-muscle invasive BlCa (NMIBC), accounting for 75–80% of all new cases, characterised by frequent recurrences and eventual progression to more aggressive, deeply invasive and metastatic disease, or muscle-invasive BlCa (MIBC), an aggressive, locally invading carcinoma, corresponding to 20–25% of all cases, with propensity for metastisation [3,4]. Haematuria is the most common clinical sign of BlCa, although it also occurs in several common benign disease such as urinary tract infections and non-infectious inflammatory conditions. Presently, BlCa diagnosis generally involves cytoscopic examination, an expensive and invasive procedure, complemented by urine cytology [5,6,7]. However, the latter has limited accuracy, particularly for identification of low-grade papillary tumours, and the invasive nature of cystoscopic examination entails patient discomfort and, in some cases, infection [5]. Moreover, because of the high incidence, recurrence and progression rate, active long follow-up is required, making BlCa the costliest malignancy [8]. Thus, early, accurate and non-invasive BlCa detection is the determinant to improve both patients and healthcare financial management.

Epigenetic changes, including DNA methylation, have been largely investigated for cancer detection [9]. Owing to chemical and biological stability, DNA methylation-based biomarkers have potential clinical applications in early cancer detection, diagnosis, follow-up and targeted therapies [10]. Previously, two independent DNA methylation-based biomarker panels have been reported as promising tests for accurate early detection of BlCa [11,12]. In 2010, a three-gene panel comprised *GDF15, TMEFF2* and *VIM* methylation identified BlCa with 94% sensitivity and 100% specificity in urine samples from 51 BlCa patients [11]. More recently, a panel testing the promoter methylation of two microRNAs—*miR129-2* and *miR663a*—identified urothelial carcinoma (from upper and lower urinary tracts) with a sensitivity of 87.8% and specificity of 82.7% in 49 urine samples from patients with urothelial carcinoma [12]. Furthermore, the same panels could discriminate BlCa from other common genitourinary cancers (i.e., from kidney and prostate). Nonetheless, both studies used a singleplex approach, and the ability of these tests to discriminate BlCa from common benign conditions of the urinary tract with overlapping manifestations, especially inflammatory diseases, has not been adequately explored, thus far. Indeed, inflammatory conditions of the urinary tract may negatively impact the specificity of urinary-based biomarkers for BlCa detection, increasing false positive results and entailing unnecessary complementary invasive tests [6,13,14].

Thus, we sought to assess whether the most promising markers in each published panel—*miR-663a* (*miR663a*_me_) and *Vimentin* (*VIM*_me_)—might accurately discriminate BlCa from inflammatory conditions in voided urine, allowing for the development of a multiplex test that could be used for early detection in clinical practice.

## 2. Experimental Section

### 2.1. Patients and Tumour Sample Collection

Ninety-four primary BlCa tissue samples were obtained from a consecutive series of patients diagnosed, treated with transurethral resection (TUR) or radical cystectomy, between 1994 and 2011, and followed at Portuguese Oncology Institute of Porto (IPO Porto), Portugal (Table 1). Briefly, tumour samples were obtained during surgery and immediately snap-frozen, stored at −80 °C and subsequently macrodissected for tumours’ cells enrichment and cut in cryostat for DNA extraction. Routine collection and processing of tissue samples allowed for pathological examination, classification, grading and staging [15]. For control purposes, an independent set of 19 normal bladder mucosae (NB) samples were also collected from BlCa-free individuals (prostate cancer patients submitted to radical prostatectomy) (Table 1).

### 2.2. Urine Sample Collection and Processing

For the “Testing sets”, 27 voided urine samples (one per patient) were collected from BlCa patients, diagnosed and treated between 2006 and 2016 at IPO Porto, as well as a set of 24 voided urine samples from healthy donors (HD), also from IPO Porto, with no personal or familial history of cancer, used as controls (Table 1). The “Validation sets” comprised: (1) 100 urine samples from BlCa patients, diagnosed and treated between 2002 and 2016 at IPO Porto, and 57 urine samples from HD collected at IPO Porto, and (2) an independent set of control urine sediments (*n* = 174) from patients diagnosed with urinary tract inflammatory conditions (IC), diagnosed between 2008 and 2014 at the University Hospital of Cordoba (UHC). All BlCa patients’ urines were obtained before treatment. Moreover, all sets of samples were collected from different cohorts of patients. Informed consent was obtained from patients and controls after approval from the ethics committees of IPO Porto and UHC (CES-IPO 019/08, approval date: 16th January 2008). All urine samples were processed by immediate centrifugation at 4000 rpm for 10 min; the respective pellet was washed twice with phosphate-buffered saline (PBS) and stored at −80 °C.

### 2.3. Nucleic Acids Isolation, Bisulfite Modification and Multiplex qMSP Analysis

DNA was extracted from frozen BlCa and NB tissues, and all urine sample sets, using a standard phenol-chloroform protocol [16], and its concentration determined using a Qubit 3 Fluorometer (Thermo Fisher Scientific, Waltham, MA, USA). Bisulfite modification was performed through sodium bisulfite, using the EZ DNA Methylation-Gold™ Kit (Zymo Research, Irvine, CA, USA), according to manufacturer’s protocol. For this, 1000 ng and 50 ng of DNA were converted for tissues and urine sediments, respectively. Quantitative methylation levels were performed using Xpert Fast Probe Master Mix (GRiSP, Porto, Portugal), and multiplex reactions were run in triplicates in 96-well plates using an Applied Biosystems 7500 Sequence Detector (Perkin Elmer, Waltham, CA, USA), with Beta-Actin (ACTB) as internal reference gene for normalization. Primer and probe sequences were designed using Methyl Primer Express 1.0 and purchased from Sigma-Aldrich (St. Louis, MO, USA) (Supplementary Table S1). Additionally, six serial dilutions (dilution factor of 5×) of a fully methylated bisulphite modified universal DNA control were included in each plate to generate a standard curve. In each sample and for each gene, the relative DNA methylation levels were determined using the following formula: ((target gene/ACTB) ×1000). A run was considered valid when previously reported criteria were met [11].

### 2.4. Statistical Analysis

Differences in quantitative methylation values were assessed with the non-parametric Mann-Whitney *U* (MW) and Kruskal-Wallis (KW) tests. Associations between age, gender, grade, invasion of muscular layer and methylation levels were carried out using Spearman’s correlation, MW or KW tests, as appropriate. For multiple comparisons, Bonferroni’s correction was applied in pairwise comparisons.

Biomarker performance parameters, including sensitivity, specificity, positive predictive value (PPV), negative predictive value (NPV), accuracy and positive and negative likelihood ratios (LR), were estimated [17]. Receiver operator characteristics (ROC) curves were constructed by plotting the true positive (sensitivity) against false positive (1-specificity) rate, and the area under the curve (AUC) was calculated. The higher value obtained from the sum of sensitivity and 1-specificity in each ROC-curve was used as cut-off to categorise samples as methylated or non-methylated. ROC curves were constructed using logistic regression model for DNA methylation panel. Disease-specific and disease-free survival curves (Kaplan-Meier with log rank test) were computed for standard variables and for categorised genes’ promoter methylation status. A Cox-regression model comprising all significant variables (univariable and multivariable model) was computed to assess the relative contribution of each variable to the follow-up status. All two-tailed *p* values were derived from statistical tests, using a computer-assisted program (SPSS Version 26.0, IBM, Armonk, NY, EUA) and the results were considered statistically significant at *p* < 0.05. Bonferroni’s correction for multiple comparisons was used when applicable.

## 3. Results

### 3.1. Methylation Analysis and Performance of the Multiplex Panel in BlCa Tissue Series

**Figure 1:**
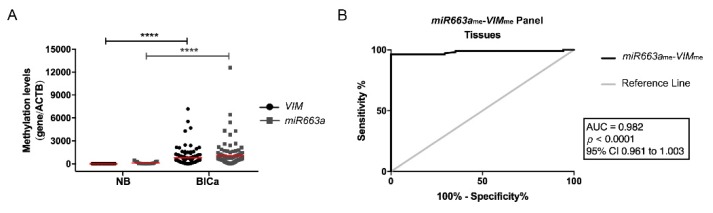
(**A**) Distribution of *VIM*_me_ and *miR663a*_me_ levels in normal bladder mucosae (NB; *n* = 19) and bladder carcinoma (BlCa; *n* = 94) tissue samples. Mann-Whitney U test, **** *p* < 0.0001. Median is represented by the red line. (**B**) Receiver operator characteristic (ROC) curve evaluating the performance of the *VIM*_me_-*miR663a*_me_ panel for the identification of BlCa in tissue samples. (AUC—Area under the curve; CI—Confidence interval; ACTB—Beta-Actin; VIM—Vimentin).

To confirm the previously published performance of *miR663a* and *VIM* promoter methylation as BlCa biomarkers, tissue samples were tested. As expected, both *miR663a* and *VIM* were found hypermethylated (76.6% and 94.4%, respectively) in most BlCa tissue samples, and methylation levels were significantly higher compared to NB (*p* < 0.0001 and *p* < 0.0001, respectively) (Figure 1A). The two genes independently performed well as BlCa detection biomarkers in tissues, with an AUC of 0.979 for *VIM*_me_ (95% confidence interval (CI): 0.956–1.002, *p* < 0.0001), and of 0.897 for *miR663a*_me_ (95% CI: 0.836–0.959, *p* < 0.0001). Moreover, in combination as multiplex panel, it accurately discriminated BlCa from NB with 96.3% sensitivity and 88.2% specificity, corresponding to an AUC of 0.982 (Figure 1B; Supplementary Table S2).

### 3.2. Methylation Analysis and Performance of Multiplex Panel in BlCa Testing Set

**Figure 2:**
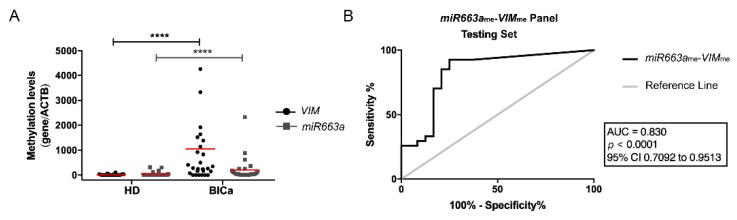
(**A**) Distribution of *VIM*_me_ and *miR663a*_me_ levels in the Testing Cohort, composed by healthy donors (HD; *n* = 24) and bladder carcinoma (BlCa; *n* = 27) urine samples. Mann-Whitney U test, **** *p* < 0.0001. Median is represented by the red line. (**B**) Receiver operator characteristic (ROC) curve evaluating the performance of the *VIM*_me_-*miR663a*_me_ panel for the identification of BlCa in urine samples of the Testing Cohort. (AUC—Area under the curve; CI—Confidence interval; ACTB—Beta-Actin; VIM—Vimentin).

Paralleling the previous observations in tissues, *miR663a*_me_ and *VIM*_me_ levels were significantly higher in BlCa urine samples than in those of controls (*p* < 0.0001 and *p* < 0.0001, Figure 2A), and the multiplex panel discriminated BlCa from HD with 92.6% sensitivity and 90% NPV (Supplementary Table S2), corresponding to an AUC of 0.83 (Figure 2B).

### 3.3. Methylation Analysis and Performance of VIM_me_ and miR663_me_ Multiplex Panel for BlCa vs. HD

**Figure 3:**
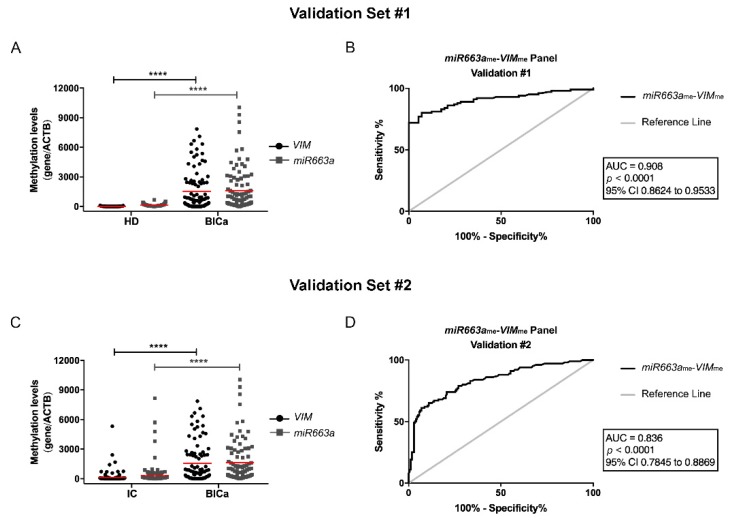
(**A**) Distribution of *VIM*_me_ and *miR663a*_me_ levels in the Validation Cohort #1, composed by healthy donors (HD; *n* = 57) and bladder carcinoma (BlCa; *n* = 100) urine samples. Mann-Whitney U (MW) test, **** *p* < 0.0001. Median is represented by the red line. (**B**) Receiver operator characteristic (ROC) curve evaluating the performance of the *VIM*_me_-*miR663a*_me_ panel for the identification of BlCa in urine samples of the Validation Cohort #1. (**C**) Distribution of *VIM*_me_ and *miR663a*_me_ levels in the Validation Cohort #2, composed by inflammatory controls (IC; *n* = 174) and bladder carcinoma (BlCa; *n* = 100) urine samples. MW test, **** *p* < 0.0001. (**D**) ROC curve evaluating the performance of the *VIM*_me_-*miR663a*_me_ panel for the identification of BlCa in urine samples of the Validation Cohort #2. (AUC—Area under the curve; CI—Confidence interval; ACTB—Beta-Actin; VIM—Vimentin).

In line with the testing set results, a higher number of malignant samples disclosed significantly higher *VIM*_me_ and *miR663*_me_ levels than HDs (*p* < 0.0001 and *p* < 0.0001, respectively) in the validation sets (Figure 3A). ROC curve analysis confirmed a high discriminative ability of *VIM*_me_-miR663_me_ panel, with an AUC of 0.91 (Figure 3B). Indeed, the multiplex panel discriminated BlCa from HD subjects with 87% sensitivity and 86% specificity (Table 2).

**Figure 4:**
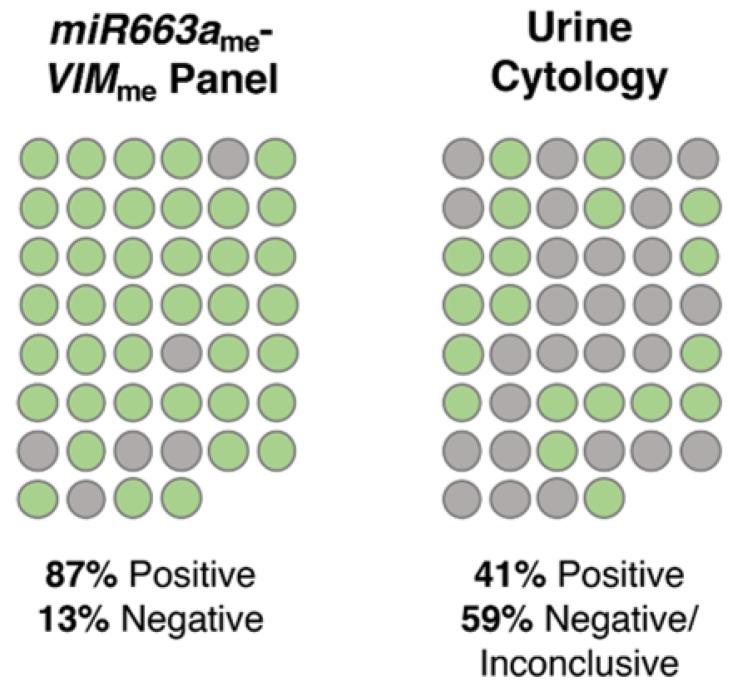
Representation of the percentage of bladder cancer (BlCa) cases correctly identified with the *VIM*_me_-*miR663a*_me_ panel and a standard urine cytology analysis. Green circles represent positive cases, grey circles represent negative/inconclusive cases.

Remarkably, the proportion of true positive cases detected by the *VIM*_me_-miR663_me_ multiplex panel was significantly higher than that of urine cytology (*p* < 0.001). Indeed, of 46 BlCa cases with valid urine cytology results, only 19 were classified as positive, 17 as negative and 10 as “inconclusive/suspicious”, corresponding to 41% sensitivity (Figure 4). Contrarily, the *VIM*_me_-miR663_me_ multiplex panel correctly identified 40/46 cases as BlCa, corresponding to an overall sensitivity of 87% (Figure 4). Importantly, 12 of 14 low-grade papillary carcinomas were accurately identified by *VIM*_me_-*miR663*_me_ multiplex panel, whereas cytology merely identified four cases.

### 3.4. Methylation Analysis and Performance of VIM_me_ and miR663_me_ Multiplex Panel for BlCa vs. IC

In urine samples, *VIM*_me_-*miR663*_me_ levels discriminated BlCa from IC patients (Figure 3C), with 80% sensitivity, 75.3% specificity and, importantly, 86.8% NPV (Table 2), corresponding to an AUC of 0.836 (Figure 3D). Remarkably, a 2.86 Positive LR and a Negative LR of 0.21 were also disclosed by *VIM*_me_-*miR663*_me_ multiplex panel in this setting.

### 3.5. Clinicopathologic Correlations and Survival Analyses

High-grade papillary BlCa showed significantly higher *miR663a*_me_ levels than low-grade papillary BlCa (*p* = 0.007), in tissue samples. The same was observed in urine samples from the validation set (*p* = 0.0072), a result which was extensive to *VIM*_me_ (*p* = 0.0052) (Supplementary Figure S1). No additional associations were disclosed between *VIM*_me_ and *miR663a*_me_ levels and other standard clinical variables, including patients’ age and gender.

Follow-up data was available for 91 (out of 94) IPO Porto’s BlCa patients that provided tissue samples. The median follow-up time was 66 months (range: 1–203 months). At the last follow-up timepoint, 30 patients were alive with no evidence of cancer, 12 patients were alive with disease, 29 had deceased due to BlCa and 23 died from other causes. Univariable and multivariable Cox regression analysis were performed, including the variables grade, invasion of muscular layer, gender and age. As expected, a poor outcome was depicted for patients with higher grade and muscle invasive BlCa (*p* = 0.001 and *p* < 0.0001, respectively) (Table 3). In the multivariate model for disease-specific survival, *miR663a*_me_ levels, higher grade and muscle invasion were independent predictors of outcome (*p* = 0.04, *p* = 0.035 and *p* = 0.031, respectively; Table 3). Moreover, after categorization into NMIBC *vs.* MIBC, tumours with higher *miR663a*_me_ levels implied a 3.7-fold increased risk of cancer-related death among patients with MIBC (95% CI: 1.32–10.25, *p* = 0.013; Supplementary Figure S2). Contrarily, no associations were found for *miR663a*_me_ or *VIM*_me_ levels concerning disease-free survival.

## 4. Discussion

Bladder cancer is a major health concern worldwide, with an expected significant increase in incidence and mortality within the next two decades [1,2]. Early detection is critical for adequate management, aiming to reduce disease-specific mortality, as well as the economic burden imposed by BlCa treatment and follow-up. Because currently available diagnostic tools require invasive examination [13,14], development of non-invasive and less costly tests for early detection and monitoring are likely to have a significant impact in clinical practice. Although several molecular biomarkers, including epigenetic-based, have been developed for that end, discrimination of BlCa from other urinary tract malignancies and, more importantly, from benign conditions causing haematuria, including inflammatory diseases, remains a challenge. Indeed, most control samples used in biomarker discovery studies, including our own, mostly comprise normal/healthy donors, disregarding the fact that a biomarker-based test would be offered to an “at-risk” population, including patients experiencing suspicious symptoms. Therefore, based on two previously published studies by our research team [11,12], we tested whether a *miR663a*_me_ and *VIM*_me_ multiplex panel could accurately discriminate BlCa from normal individuals and those afflicted with inflammatory conditions of the genitourinary tract.

Because both *miR663a*_me_ and *VIM*_me_ were previously assessed using two different “simplex” multi-gene biomarker panels, we firstly tested *miR663a*_me_ and *VIM*_me_ in multiplex in a consecutive series of primary BlCa tissue samples and normal urothelial mucosae to confirm those previous results. Indeed, employing a multiplex reaction allows for downscaling the initial tissue/body fluid sample requirements, but also the quantity of DNA required for each test [18]. Remarkably, as expected, the *miR663a*_me_-*VIM*_me_ multiplex panel discriminated BlCa from NB tissues with high sensitivity and specificity (96.3% and 88.2%, respectively), confirming the previous observations for the two markers separately [11,12]. In urine samples from the testing set, although the performance of the multiplex panel was slightly inferior to that of tissues, 92.6% sensitivity and 90% NPV was reached. Indeed, it should be recalled that a relatively small number of cancer cells are exfoliated into urine, which are subsequently “diluted” among a larger population of normal-looking urothelial cells. Thus, the tumour DNA content in urine is actually minute [19] and sensitivity over 90% should be regarded as a very encouraging result. Furthermore, in the validation set, comprising a larger independent cohort, specificity of the *miR663a*_me_-*VIM*_me_ multiplex panel increased to 86%, further increasing the potential usefulness of the test.

It should be emphasised, however, that the foremost aim of this study was to assess the multiplex panel ability to discriminate BlCa from IC, since this panel is envisaged to be tested in an “at-risk” population, including individuals complaining of haematuria, many of which will be found to harbour urinary tract inflammatory conditions. Although, in this setting, sensitivity and specificity were slightly reduced, NPV increased (86.8%), which is an important finding [20]. Indeed, it is expected that among tested individuals, most will not have a neoplastic condition and, thus, the higher the NPV, the larger the proportion of those subjects that will not be submitted to confirmatory, invasive, procedures, supporting the good performance of the test in discriminating patients negative for malignant condition. Importantly, an LR (+) of 2.86 and an LR (−) of 0.21 values were observed, indicating that a negative result decreases by 30% the probability of misdiagnosis [17].

Despite the fact that several studies suggest various genomic mutations and/or proteins’ expression deregulation as biomarkers for BlCa detection and prognostication [21], the search for novel epigenetic biomarkers, mostly DNA methylation-based, for BlCa detection has been attempted by several research teams, probably due to the stability of the markers and the possibility of high-throughput tests. Although some of those previous studies report an apparently superior performance to the panel reported herein, it should be recalled that in most cases the patients’ series were smaller, only healthy donors were included as controls or these were comprised of a mixed group of healthy donors and patients with diverse urological diseases, and/or did not use a multiplex approach, which might impact in sample availability, testing time length and cost [22,23,24,25,26,27,28]. Roperch et al. proposed a three gene multiplex methylation panel (*HS3ST2*, *SEPTIN9* and *SLIT2*) combined with *FGFR3* mutations assessment, age and smoking-status at time of diagnosis in a multivariate model, for diagnosis of NMIBC in urine samples, disclosing 97.6% sensitivity and 84.8% specificity, in a smaller control cohort [29]. Nonetheless, this strategy might be more difficult to implement in clinical practice, since it requires both mutation and methylation analyses, in which the multiplex is performed in two distinct gene duplex reactions. Similarly, Dahmcke et al. proposed a six gene methylation panel (*SALL3*, *ONECUT2*, *CCNA1*, *BCL2*, *EOMES* and *VIM*) combined with the mutational analysis of *TERT* and *FGFR3*, for early detection of BlCa, in urine samples, comparing BlCa patients and patients with gross haematuria [30]. Although this panel disclosed higher sensitivity (97%), specificity was similar (76.9%) [30], and, once again, our test uses a single technique in a single reaction, requiring less amount of sample, enabling shorter response time, reduced technical skills and lower cost.

**Figure 5:**
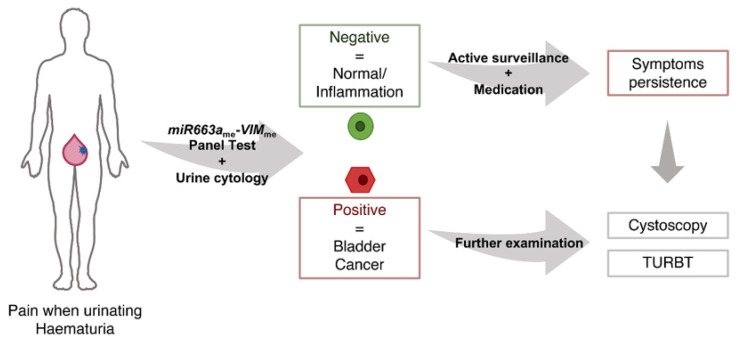
Proposed algorithm for the combination of urine cytology and *VIM*_me_-*miR663a*_me_ panel as a first-line diagnostic tests in patients with common urinary complaints. (TURBT—Transurethral Resection of Bladder Tumour).

Although urine cytology and UroVysion^TM^ fluorescence in situ hybridization (FISH) assay are the two most commonly used urine-based tests in daily practice, they present important limitations. On one hand, UroVysion^TM^ presents a not-negligible rate of false positive results; on the other hand, urine cytology has limited accuracy, especially in low grade tumours detection [6,31,32]. Although no direct comparison can be done with UroVysion^TM^, the 91.6% PPV obtained for the multiplex panel clearly demonstrates higher accuracy in identifying true positive BlCa cases. In the present study, urine cytology reached 41% sensitivity, which was easily surpassed by the 86% displayed by *miR663a*_me_-*VIM*_me_ multiplex panel. Notwithstanding, urine cytology remains an easy-to-perform and informative test, as it allows pathologists to have the first look at exfoliated neoplastic cells in urine. Having that in mind, we propose an algorithm where a urine cytology and the *miR663a*_me_-*VIM*_me_ multiplex panel could be combined as first-line diagnostic tests in patients with common urinary complaints, with the ultimate goal of reducing the number of unnecessary cystoscopies, which are invasive, uncomfortable and costly procedures (Figure 5).

In this work, we further explored the prognostic ability of the gene methylation markers, aiming to strengthen its clinical potential. Interestingly, survival analysis revealed that high *miR663a*_me_ levels independently predicted poor disease-specific survival in BlCa patients, especially those with MIBC. Thus, the *miR663a*_me_-*VIM*_me_ multiplex panel not only conveys diagnostic, but also prognostic information.

Taking into account the promising results obtained, unveiling the putative biological relevance of *miR663a* and *VIM* promoter methylation in bladder carcinogenesis may provide new important insights. VIM encodes for vimentin, an intermediate filament characteristic of cells with mesenchymal phenotype, not expressed in most normal epithelia (including urothelium), nor in most carcinomas [33]. *VIM* de-novo expression or overexpression has been reported in various epithelial cancers, including those of prostate [34], breast [35] and lung [36], associating with increased tumour growth and invasion. In these instances, vimentin expression has been associated with epithelial to mesenchymal transition (EMT), a biological process associated with tumour invasiveness [33]. Although *VIM* promoter methylation has been proposed as a detection and/or prognostic marker for other malignancies, biological functions are yet to be truly explored. Moreover, microRNAs have been extensively implicated in urological malignancies [37]. Interestingly, a dual role has already been described for miR663a, having a tumour suppressive activity in thyroid carcinoma [38] and glioblastoma [39], whereas an oncogenic function was reported in prostate cancer [40] and osteosarcoma [41]. Additionally, miR663a’s downregulation fostered cell proliferation by JunD overexpression in small-cell lung carcinoma [42], and HMGA2 in hepatocellular carcinoma [43], while Transforming Growth Factor-1 (TGF-β1) [44] overexpression was linked with invasion in the tumour type. Nevertheless, it should be recalled that not all biomarkers require to have a relevant biological role in tumorigenesis.

Importantly, to assure accuracy and validity of the proposed methylation multiplex test, additional validation by others, with larger sets of samples from prospectively collected data (from both BlCa and inflammatory conditions) is warrant.

## 5. Conclusions

In summary, we demonstrated that a *miR663a*_me_-*VIM*_me_ multiplex panel accurately identifies BlCa, allowing for precise identification of this common neoplasm in urine samples. Importantly, it also discriminates BlCa patients from those with urinary tract inflammatory conditions, although with inferior performance comparatively to healthy subjects. Thus, the implementation of this panel might assist clinicians in better stratifying patients for confirmatory, invasive examinations, ultimately improving the cost-effectiveness of BlCa diagnosis and management. Moreover, in the same analysis, *miR663a*_me_ analysis would identify patients at higher risk for cancer progression, further highlighting the promise of this panel for patient monitoring.

## Figures and Tables

**Table 1:** Clinical and histopathological characteristics of patients with bladder carcinoma (BlCa), normal bladder mucosae (NB), healthy donors (HD) and inflammatory controls (IC).

	Tissues		Urines				
			Testing Set		Validation Sets		
Clinicopaphological Features	Bladder UC	Normal Bladder Mucosae	Bladder UC	Healthy Donors	Bladder UC	Healthy Donors (#1)	Inflammatory Controls (#2)
Patients, *n*	94	19	27	24	100	57	174
Gender, *n*							
Males	78	19	20	13	79	16	132
Females	16	0	7	12	21	41	42
Median age, yrs (range)	69 (45–91)	63 (48–75)	69 (47–88)	45 (39–61)	68 (38–91)	49 (41–64)	64 (18–92)
Grade, *n*							
Papillary, low-grade	34	n.a.	13	n.a.	51	n.a.	n.a.
Papillary, high-grade	33	n.a.	8	n.a.	26	n.a.	n.a.
Invasive, high-grade	27	n.a.	6	n.a.	23	n.a.	n.a.
Invasion of Muscular Layer, *n*							
NMIBC	67	n.a.	19	n.a.	77	n.a.	n.a.
MIBC	27	n.a.	8	n.a.	23	n.a.	n.a.

#1—Validation Set #1; #2—Validation Set #2; yrs—years; n.a.—non applicable; NMIBC—Non-Muscle Invasive Bladder Cancer; MIBC—Muscle Invasive Bladder Cancer, UC—Urothelial Carcinoma.

**Table 2:** Performance of *VIM*_me_-*miR663a*_me_ panel for the detection of bladder cancer in Validation Cohorts #1 and #2. (PPV—positive predictive value; NPV—negative predictive value).

Samples	Biomarker Performance	*miR663a*_me_-*VIM*_me_ (%)
Validation #1	Sensitivity	87.0
	Specificity	86.0
	PPV	91.6
	NPV	79.0
	Accuracy	86.6
Validation #2	Sensitivity	80.0
	Specificity	75.3
	PPV	65.0
	NPV	86.8
	Accuracy	77.0

PPV—Positive Predictive Value; NPV—Negative Predictive Value.

**Table 3:** Cox regression models assessing the potential of clinical and VIMme and miR663ame levels in the prediction of disease-specific survival for bladder carcinoma (BlCa) patients.

Disease-specific Survival	Variables	Hazard Ratio (HR)	95% CI for OR	*p*
Univariate	Invasion of muscular layer	6.15	2.76–13.72	0.0001
	Grade			
	PLG vs. PHG	15.59	2.03–119.94	0.008
	PLG vs. IHG	32.83	4.31–250.06	0.001
	Age	2.34	0.98–5.59	0.060
	Gender	1.02	0.39–2.70	0.970
	miR663a methylation ≤ median	1.61	0.75–3.48	0.225
	VIM methylation ≤ median	1.07	0.50–2.28	0.861
Multivariate	Invasion of muscular layer	3.54	1.12–11.19	0.031
	Grade			
	PLG vs. PHG	8.03	0.97–66.32	0.053
	PLG vs. IHG	11.89	1.18–119.37	0.035
	miR663a methylation ≤ median	2.67	1.05–6.81	0.040
	VIM methylation ≤ median	1.12	0.51–2.42	0.783

CI—confidence interval; OR—odds ratio; PLG—papillary low-grade; PHG—papillary high-grade; IHG—invasive high-grade.

## Supplementary Materials

The following are available online at https://www.mdpi.com/2077-0383/9/2/605/s1, Figure S1: Distribution of *miR663a*_me_ and *VIM*_me_ levels in bladder carcinoma tissue samples categorised by grade; Figure S2: Kaplan-Meyer curves representing disease-specific survival according to *miR663a*_me_ status; Table S1: Sequences of the primers and probes used in the quantitative methylation-specific PCR experiments; Table S2: Performance of *VIM*_me_, *miR663a*_me_ and *VIM*_me_-*miR663a*_me_ panel for the detection of bladder cancer in Tissues and Testing Set.
