# Supplementary material for: A Multiplex Test Assessing MiR663ame and VIMme in Urine Accurately Discriminates Bladder Cancer from Inflammatory Conditions
Source: J Clin Med. 2020 Feb 24;9(2):605. doi: 10.3390/jcm9020605 (PMC7073678; doi:10.3390/jcm9020605)
Supplement: Supplementary file 1 [file jcm-09-00605-s001.pdf]

Supplementary Material – Monteiro-Reis *et al*, Journal of Clinical Medicine, 2020

Supplementary Table S1 – Sequences of the primers and probes used in the quantitative methylation-specific PCR experiments.

Primer Set		Sequence	Product size, bp	$T_{Annealing}$ ºC
ACTB_QMSP	Forward Primer	5′ TGGTGATGGAGGAGGTTTAGTAAG T 3′	133	60
	Reverse Primer	5′ AACCAATAAAACCTACTCCTCCCTTAA 3′		
	Probe	5′ CY5 - ACCACCACCCAACACACAATAACAAACACA - MGB 3'		
miR663a_QMSP	Forward Primer	5′ GGGATAGCGAGGTTAGGTC 3′	101	60
	Reverse Primer	5′ CATTCGTAACGAATAAAACCC 3′		
	Probe	5' VIC - CTCTTCCTCGCCTACG - MGB 3'		
VIM_QMSP	Forward Primer	5′ TTCGGGAGTTAGTTCGCGTT 3′	108	60
	Reverse Primer	5′ ACCGCCGAACATCCTACGA 3′		
	Probe	5' FAM - TCGTCGTTTAGGTTATCGT - MGB 3'		

Supplementary Table S2 – Performance of VIM_{me}, $miR663a_{me}$, and VIM_{me}-$miR663a_{me}$ panel for the detection of bladder cancer in Tissues and Testing Cohort (urines). (PPV – positive predictive value; NPV – negative predictive value)

Samples	Biomarker Performance	VIM_{me} (%)	$miR663a_{me}$ (%)	$miR663a_{me}$-VIM_{me} (%)
**Tissues**	Sensitivity	94.4	76.6	96.3
	Specificity	94.1	94.1	88.2
	PPV	99.0	98.8	98.1
	NPV	72.7	39,0	78.9
	Accuracy	94.4	79.0	95.2
**Testing Set (Urines)**	Sensitivity	78.6	82.1	92.6
	Specificity	96.4	82.1	75.0
	PPV	97.1	87.3	80.6
	NPV	75.0	75.4	90.0
	Accuracy	85.7	82.1	84.3

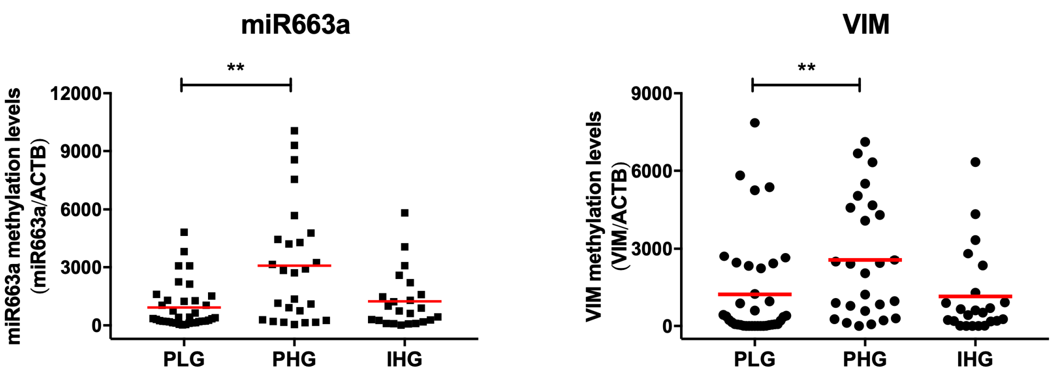

Supplementary Figure S1 – Distribution of *miR663a*me and *VIM*me levels in bladder carcinoma (BlCa; n=94) tissue samples categorized by grade. Mann-Whitney U test, **P<0.01. (PLG – Papillary Low Grade; PHG - Papillary High Grade; IHG – Invasive High Grade).

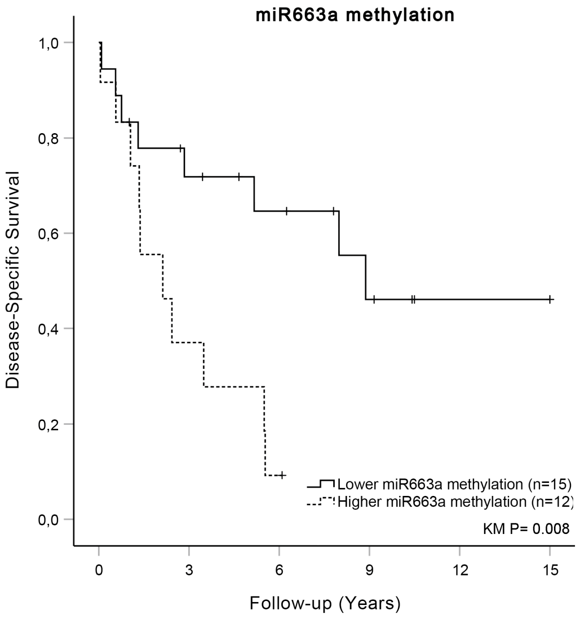

Supplementary Figure S2 – Kaplan-Meyer (KM) curves representing disease-specific survival according to *miR663a*me status.
